# Editorial: Immunoregulatory Mechanisms of Interferon

**DOI:** 10.3389/fimmu.2020.00187

**Published:** 2020-02-14

**Authors:** Claudia U. Duerr, Jörg H. Fritz

**Affiliations:** ^1^Institute of Microbiology, Infectious Diseases and Immunology, Charité – University Medical Centre Berlin, Berlin, Germany; ^2^Department of Microbiology & Immunology, McGill University, Montreal, QC, Canada; ^3^McGill University Research Centre on Complex Traits (MRCCT), McGill University, Montreal, QC, Canada; ^4^FOCiS Centre of Excellence in Translational Immunology (CETI), McGill University, Montreal, QC, Canada; ^5^Department of Physiology, McGill University, Montreal, QC, Canada

**Keywords:** interferon, immunmodulation, immunity, homeostasis, infection

Interferons (IFN) belong to the family of cytokines and have been described first in the late 1950s as an inhibitory factor of viral replication (1, 2). Since then, the impact of interferon has been greatly expanded and its function comprises a role not only in different types of infection, cancer and autoimmunity but importantly also in immune homeostasis. IFN have important anti-viral and anti-bacterial effects but it is becoming more and more evident that they are true immunomodulators and have a major impact on the development and maintenance of innate and adaptive immunity. IFN are classified into three groups: type I (IFN-I), type II (IFN-II, IFN-γ), and type III (IFN-III or IFN-λ). IFN can act in an autocrine and paracrine fashion upon induction by pattern recognition receptors (PRRs) sensing viral and bacterial components as well as danger associated molecular patterns (DAMPs).

This Research Topic features several **Review and Original Research articles** as well as one **Hypothesis and Theory article** on the different facets of interferon: evolution of IFN, signal transduction, role of IFN in infections, impact of IFN on metabolism and its effect on homeostasis and immune responses at barrier surfaces. Articles focus on all three types of IFN giving an important overview of current concepts of IFN functionality and emphasize the significance of IFN in immunity and beyond.

## Evolution of IFN and IFN in Fish and Birds

IFN and thereby genes for interferon are present not only in mammals but exist already in cartilaginous fish and bony fish. Secombes and Zou review the evolution of the interferon system by comparing interferon encoding genes of cartilaginous fish using mainly elephant shark and bony fish as an example with the ones in mammals. Genes for interferon and interferon receptors are present in elephant shark as well as orthologs genes of Toll like receptors (TLRs) as sensors and genes for components of IFN signal transduction emphasizing the key role and importance of interferon throughout evolution. Next to TLRs, RIG-I-like receptors comprise another important group of PRRs which are able to induce interferon expression. In the fresh water fish Grass carp, *Ctenopharyngodon Idella*, Rao et al. report that the RLR laboratory of genetics and physiology (LGP2) acts as a negative regulator of RIG-1 and MDA-5. Inhibition of RIG-1 and MDA-5 signal transduction is regulated by suppression of ubiquitination motifs as well as interference of LGP2 with interferon regulatory factors (IRFs). In an original research paper, Ding et al. describe two novel types of IFN-I in fish, specifically in Perciforme Fish Large Zellow Croaker *Larimichthys crocea* (*L. crocea*), namely IFN-δ and IFN-η. Sequence and phylogeny analysis of fish emphasizes the distinct IFN-I sequence motif. IFN-δ and IFN-η are induced in several different organs of *L. crocea* and are induced upon viral challenge. Moreover, fish cells respond to the novel IFN with the induction of interferon induced genes (ISGs). This work reports two novel IFN-I forms and highlights the importance of IFN for fish immunity. IFN were discovered because of their antiviral activity. These early experiments were done using influenza virus and fragments of chicken chorio-allantoic membrane (1) brought to our attention by Santhakumar et al. who provide a detailed overview of avian interferon and the induction of IFN by PRRs in birds. Interestingly, retinoic acid-inducible gene I (RIG-I), which is an important sensor in the mammalian IFN system is missing in *Galliformes* including chicken and turkeys which might influence their susceptibility to RNA viruses. Other sensors including TLRs and components involved in signal transduction are further compared and discussed and highlight thereby the similarities and differences between the mammalian and avian interferon system.

## Signal Transduction of IFN

Upon ligation of IFN receptors, signal is further transduced by a complex signaling cascade. Majoros et al. provide a conceptual framework on IFN induced signaling pathway by summarizing and discussing current knowledge of the canonical and non-canonical IFN signaling. The canonical IFN pathway involves signal transducer and activator of transcription (STAT) tyrosine phosphorylation by receptor bound activated Januse kinase (JAK) as well as STAT1 homodimers and the induction of IFN stimulated genes (ISGs) in contrast to the non-canonical IFN pathway which includes kinase dead JAKs. Recent evidence indicates that IFN is also able to influence signaling pathways not directly linked to the interferon system. Kopitar-Jerala presents a detailed overview of crosstalks between the IFN and the inflammasome system such as the regulation of expression of Caspase-11 by IFN. Bachmann et al. report a synergistic activation of inducible nitric oxid synthase (iNOS) by IFN-I in combination with IL-1β/TNF in hepatocytes. iNOS expression is also dependent in the liver on IFNAR1 in an acute liver injury model.

## Role of IFN in Viral, Bacterial and Parasitic Infections

IFN are key modulators of immunity in infections. Schulz and Mossman discuss novel findings to counteract interferon responses upon viral infection. The steps of interferon induction and signaling are highlighted and the respective viral strategies are discussed including TLRs, RLRs, and IFNAR regulation and signaling. Recognizing viral RNA structures by the receptor RIG-1 is essential in the induction of interferon upon viral infection. Liu et al. provide a detailed overview of the signal transduction of RIG-1 as well as the importance of post-translational modification. This review further highlights recent insights into viral mechanisms of evasion to avoid host detection by interfering with the detection by RIG-1 and complements thereby the previous mentioned review. IFN-I has been identified due to its antiviral activity. Murira and Lamarre summarize in a mini review the role of IFN-I in chronic infections and discuss the role of IFN-I in immunopathology in different virally induced chronic diseases. The ubiquitous receptor expression of IFN-I is highlighted here which may be responsible for prolonged expression and sensing in chronic infections. This review article is complemented by an original research paper by Daugan et al. deciphering the importance of prolonged IFN-I sensing and its link to antigen specificity in chronic lymphocytic choriomeningitis virus (LCMV) infection. In addition to viral infections, IFN-I is key in bacterial infections and can be here beneficial or detrimental for the host. This essential role of IFN-I in bacterial infection is emphasized by Kovarik et al. Many PRRs are activated by bacterial components and can thereby also induce IFN. Deficiency of IFNAR1 influences susceptibility to bacterial infections, however, it is also important whether other IFN such as IFN-II is present or how IFNAR1 is regulated upon bacterial infection. This review highlights thereby the importance of a balance within the different groups of IFN and discusses functions of IFN by tissue tropism. Further differences and similarities of mouse and human interferon systems are discussed. The importance of IFN in infection is not limited to viral and bacterial challenge but has been also reported for protozoan infections highlighted by Silva-Barrios and Stäger. A detailed overview of current knowledge is given of the role of IFN and infection by the parasites *Plasmodium, Leishmania, Trypanosoma* and *Toxoplasma*. Due to complex life cycles of parasites and thereby different influences on the immune system, IFN can also play here a beneficial or detrimental role for the host. In an original research article, Sohrabi et al. investigate the expression of interferon-induced GTPases [guanylate-binding proteins (GBPs)] focusing on Gbp2b/Gbp1 and Gbp5 in different mouse strains at steady state and upon infection with *Leishmania major*. Gbp2b/Gbp1 and Gbp5 are differently expressed in the analyzed mouse strains indicating different regulation of these genes depending on genetics as well as on the level of inflammation.

## Role of IFN in Cancer and Sepsis Treatment

Novel therapeutics against cancer are essential and involvement of IFN in anti-tumor immune responses are reviewed by Müller et al. This review highlights current literature of IFN-I (mainly α and β) and its immunomodulator function for different immune cells with the focus on NK cells and its importance in protective anti-tumor immune responses. The tumor environment has come into focus in basic research of tumor immunology but also for novel therapeutic strategies. Pylaeva et al. emphasizes the importance of IFN-I in its regulation of tumor associated neutrophils. IFN restrains the subpopulation of N2 neutrophils and thereby enhances activity and function of N1 neutrophils, which are involved in tumor growth and metastasis. Additionally, IFN is discussed as a therapeutic target in infections. Rackov et al. take this further in a mini review by reconsidering IFN-I (IFN-β) in sepsis, specifically in the delayed phase of sepsis characterized by immunosuppression.

Type III IFN (IFN-λ), the most recently identified interferon group, was discovered in 2003 (3, 4). IFN-λ is further divided into IFNλ-1, λ-2, λ-3, and λ-4. Syedbasha and Egli highlight sequence similarities within this novel interferon group as well as point out differences in signal transduction between IFN-I and IFN-III. Moreover, the link between single-nucleotide polymorphisms (SNPs) in both, IFN-λ and IFNLR genes, and the clinical outcome of viral induced hepatitis are emphasized. In a complementary review, Boisvert and Shoukry highlight specifically the clinical outcome of acute and chronic hepatitis C virus infection in regard to polymorphism in the IFNλ-3 gene. Expression of IFNλ-3 influences the replication of HCV and thus is an important factor in the immune response. Moreover, this review summarizes IFNLR expression in different cells of hematopoietic origin. In contrast to the ubiquitous expression of IFNAR1/2, IFNLR is expressed mainly on epithelial cells. Lasfar et al. review the roles of IFN-III in immunity and infection at the pulmonary and vaginal mucosa and focus on IFN-III induced epithelial immunity. In an original research article, Wang et al. report that IFN-λ (λ-1, λ-2, and λ3) have an inhibitory effect on HIV infected human macrophage cultures.

## Role of IFN at Mucosal Surfaces and Immune Homeostasis

At mucosal surfaces such as the lungs and the gut, IFN play an important role by orchestrating innate but also adaptive immunity. An appropriate immune response in response to infections is needed to ensure constant organ function. Makris et al. discuss the importance of a balanced IFN-I response in inflammation upon respiratory infections. Importantly, differences and changes of immune cells to sensitivity of IFN-I signaling in lung inflammation influences different outcomes. Moreover, the importance of IFN in pulmonary infections of viral and bacterial origin is emphasized by this review. Peteranderl and Herold highlight the interferon-TNF-related apoptosis-inducing ligand (TRAIL) signaling axis in pulmonary inflammation. Both TRAIL and IFN are induced upon viral infection, able to induce cell death and show different facets by triggering protective but also detrimental responses. The crosstalk of IFN and TRAIL is here discussed in different viral but also allergic pulmonary inflammation.

The gut harbors a plethora of different microbial species and immune responses must be well-balanced to eradicate pathogens but tolerate commensal bacteria. Kotredes et al. summarize the importance of IFN in intestinal homeostasis and inflammation. Since inflammation is also able to trigger cancer, the role of IFN in mouse models of colitis but also in human inflammatory bowel diseases are highlighted. In an original research article, Kawashima et al. identify that dsRNA of lactic acid bacteria (LAB) belonging to the small intestinal commensal flora induces IFN-I and IL-12 in human dendritic cells and thereby trigger a typical type I immune response with induction of IFN-II.

Pott and Stockinger review in detail the role of IFN-I and IFN-III (IFN-λ) in bacterial and viral infections in the gut. IFN-I is protective in enteric viral in contrast to bacterial infections. The cell tropism of the IFNLR is highlighted specifically in regard to intestinal infections in contrast to IFNAR1/2 expression, which impacts on cellular activity and infection outcome.

## IFN and Metabolism

IFN are pleiotropic cytokines and it is becoming more and more evident that IFN are key in cellular and whole body metabolism. The review by Robertson and Ghazal commence with a retrospect reporting transient hypercholesterolemia upon interferon treatment. Further the molecular regulation of lipids including oxysterol by interferon upon infection and the impact of lipid regulation on immune cells are discussed. The link between interferon and lipid metabolism is poorly studied. In a hypothesis and theory article, Newmark et al. derive elegantly the hypothesis that the evolution of interferon driven immunity was driven by sterol metabolites. Fritsch and Weichhart review in detail the effect of IFN on cellular metabolism highlighting that an interferon induced state upon viral infection and the immune response are highly dependent on changes in metabolic pathways.

Collectively, our Research Topic highlights that IFN are pleiotropic cytokines important for the defense against infections but additionally crucial in many essential biological mechanisms and functions. Research of IFN in all fields of medical sciences is today more active and fascinating than ever.

## Author Contributions

All authors listed have made a substantial, direct and intellectual contribution to the work, and approved it for publication.

### Conflict of Interest

The authors declare that the research was conducted in the absence of any commercial or financial relationships that could be construed as a potential conflict of interest.

## References

[1] Isaacs A Lindenmann J Virus interference. I. The interferon. Proc R Soc Lond B Biol Sci. (1957) 147:258–67. 10.1098/rspb.1957.0048 26297790

[2] Isaacs A Lindenmann J Valentine RC. Virus interference. II. Some properties of interferon. Proc R Soc Lond B Biol Sci. (1957) 147:268–73. 10.1098/rspb.1957.0049 26297791

[3] Kotenko SV Gallagher G Baurin VV Lewis-Antes A Shen M Shah NK. IFN-lambdas mediate antiviral protection through a distinct class II cytokine receptor complex. Nat Immunol. (2003) 4:69–77. 10.1038/ni875 12483210

[4] Sheppard P Kindsvogel W Xu W Henderson K Schlutsmeyer S Whitmore TE. IL-28, IL-29 and their class II cytokine receptor IL-28R. Nat Immunol. (2003) 4:63–8. 10.1038/ni873 12469119

